# ADCC potency assay: increased standardization with modified lymphocytes

**DOI:** 10.1186/1753-6561-5-S8-P63

**Published:** 2011-11-22

**Authors:** Laurent Bretaudeau, Véronique Bonnaudet

**Affiliations:** 1 Clean Cells, Boufféré, 85600, France

## 

For few years now, the use of monoclonal antibodies represents a significant progress in different therapeutic applications. In addition to commercialization of new products, important efforts in research and development have been made to launch new therapeutic antibodies.

Many antibodies act through a mechanism of Antibody-dependant cell cytotoxicity (ADCC). National Health Agencies recommend or require the use of biological activity assays (potency) in order to characterize those pharmaceutical products. The ADCC assay combines the 3 following elements:

• The antibody of interest that is specific to a given antigen;

• The targeted cells that express the antigen of interest at their surface;

• The effector that can trigger the lysis the targeted cell when the antibody is linked to the antigen.

As the usually met ADCC assays use Natural Killer cells, isolated from healthy donors, as effectors they are hardly reproducible (Table 1). Thus, those assays can barely be validated when lots of pharmaceutical products are released. Furthermore, of the rare NK cell lines established in culture don’t express the CD16 receptor needed for the ADCC function.

**Table 1:** Advantages and drawbacks of ADCC effectors for the validation of a standardized ADCC assay

ADCC effector types	Advantages	Drawbacks
Primary NK or PBMC, isolated from donors	Representative from the genetic diversity	Not convenient for standardization Requirement for donor genotyping Requirement to evaluate several donors for accurate comparison Exposure of operators to biohazard Significant ressources required (budget and time) for cell preparation

Modified NK cell lines	Increased suitability for standardization	The NK activity may interfere with the measurement of the ADCC-related lysis Absence of functionally qualified batches of cells Absence of biosafety qualified batches of cells To be handled as a GMO

CD16-transduced lymphocytes	Increased suitability for standardization Qualified batches available in a ready-to-use format Absence of NK activity-related background	To be handled as a GMO

**Figure 1:**
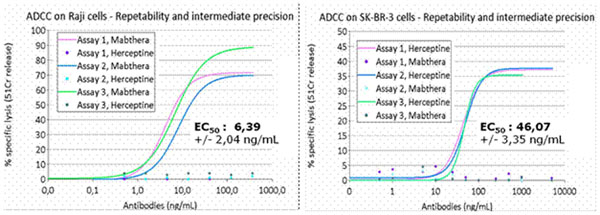
**Characterization of ADCC function of CD16-transduced lymphocytes.** Two models of antigen expressing cells were used to establish the inter-assay precision of the ADCC measurement in a standard chromium-release assay. Briefly, the cells were ^51^Cr-labelled, washed, incubated with the antibodies and the release of ^51^Cr in the supernatant was analyzed after 4h.

In this context, the use of standardized effectors should improve significantly the ADCC assays. Previous work has highlighted that human lymphocytes, modified to express the CD16 receptor, have acquired the ADCC functions [1]. Thanks to our specific know-how, clones of CD16+ lymphocytes have been produced on a large scale (10^9^ cellules). The results obtained have pointed out that cells stored in liquid nitrogen and used as soon as they were thawed, were usable for ADCC assays on a reproducible basis (Figure 1). Thanks to that approach, two models of ADCC measurement are characterized in the present presentation: CD20 and Her2neu.

The produced effector cells constitute a relevant alternative to replace the use of NK cells when the standardization of ADCC potency assay is needed.
